# Factorial invariance of the Spanish version of the PHQ-9 by gender and country in Latin America and the Caribbean

**DOI:** 10.3389/fpsyt.2025.1667612

**Published:** 2025-11-19

**Authors:** Nicole Caldichoury, Breiner Morales-Asencio, Luis Mario Castellanos-Alvarenga, Juan-Carlos Coronado, César Quispe-Ayala, Daniela Ripoll-Córdoba, Carol Saldías, Wendy Bada, Karen Alcos-Flores, Boris Zurita-Cueva, Rodrigo Duhalde-Sanhueza, Cristian Romo, Raúl Quincho-Apumayta, David Salazar, Yuliana Florez, Juan Cárdenas, Carlos Ardila-Duarte, Juan Martínez, Cesar Castellanos, Norman López

**Affiliations:** 1 Departamento de Ciencias Sociales, Universidad de Los Lagos, Osorno, Chile; 2 Consorcio Latinoamericano de Investigación (CLATI), Temuco, Chile; 3 Escuela de Psicología, Facultad de Ciencias Sociales y Comunicaciones, Universidad Santo Tomás, Temuco, Chile; 4 Departamento de Procesos Terapéuticos, Facultad de Ciencias de la Salud, Universidad Católica de Temuco, Temuco, Chile; 5 Universidad Nacional de Huancavelica, Huancavelica, Peru; 6 Escuela de Kinesiología, Facultad de Salud, Universidad Santo Tomas, Temuco, Chile; 7 Universidad Nacional Intercultural de la Amazonia, Pucallpa, Peru; 8 Departamento de Neurocirugía, Omni Hospital, Guayaquil, Ecuador; 9 Departamento de Ciencias Exactas, Universidad de Los Lagos, Osorno, Chile; 10 Universidad Nacional Daniel Alcides Carrión, Cerro de Pasco, Peru; 11 Departamento de Ciencias Sociales, Universidad de la Costa, Barranquilla, Colombia; 12 Universidad Nacional Autónoma Altoandina de Tarma, Tarma, Peru; 13 Departamento de Ciencias Básicas, Universidad Metropolitana, Barranquilla, Colombia; 14 Graduate School of Education, Ana G. Mendez University, San Juan, Puerto Rico; 15 Instituto Dominicano para el Estudio de la Salud Integral y la Psicología Aplicada (IDESIP), Santo Domingo, Dominican Republic

**Keywords:** PHQ-9, depression, factorial invariance, Latin America, structural validity, screening

## Abstract

**Introduction:**

Depression is one of the leading causes of global disease burden, particularly in regions with high inequality such as Latin America and the Caribbean (LAC). The Patient Health Questionnaire-9 (PHQ-9) is one of the most widely used instruments internationally for screening depressive symptoms, although its structural validity and diagnostic comparability in LAC still require further evidence.

**Objective:**

To examine the factorial structure, invariance by sex and country, internal consistency, and concurrent validity of the PHQ-9 in a multinational sample of Latin American adults.

**Method:**

Data from 12,124 participants across 15 LAC countries were analyzed, collected through an online form using snowball sampling. Confirmatory factor analysis (CFA) and multigroup invariance analysis were applied, along with Omega coefficient and correlations with GAD-7 and Mini-Z.

**Results:**

The PHQ-9 showed a unidimensional structure with excellent fit (CFI = .989; RMSEA = .075), adequate internal consistency (Ω = .89), and evidence of configural, metric, scalar, and strict invariance by sex and country. High correlation with GAD-7 (r = .79) and moderate correlation with Mini-Z (r = .64) supported its concurrent validity.

**Conclusion:**

The PHQ-9 demonstrated robust psychometric properties and diagnostic comparability across countries and sexes in LAC. These findings reinforce its utility as a standardized and culturally adaptable tool for screening depressive symptoms in clinical and community settings in the region.

## 1 Introduction

Depression is a mental disorder characterized by a persistently sad or irritable mood, loss of interest or pleasure, decreased energy, sleep or appetite disturbances, concentration difficulties, and feelings of worthlessness or guilt, lasting at least two weeks, with a significant impact on daily functioning (1–3).The COVID-19 pandemic exacerbated the burden of emotional disorders across all regions, with an increase in cases of major depressive disorder in the general population (4, 5). In Latin America, these effects were intensified due to inequity in access to healthcare, unemployment, food insecurity, and pressure on health systems (6).

From a clinical and public health perspective, depression is associated with multiple comorbid conditions such as insomnia (7), anxiety (8), eating problems, and suicide (9), which increase the severity of the condition and reduce therapeutic effectiveness if not detected in a timely manner (10). In addition, depression may coexist with conditions such as burnout and chronic stress, especially in contexts of work overload and lack of institutional support (11, 12). It also negatively impacts quality of life and life satisfaction, work performance, and overall individual functioning, becoming a key determinant of global disease burden (13, 14).

In this context, early detection of depression is a priority in clinical, community, and epidemiological settings. Rapid screening instruments, such as the Patient Health Questionnaire-9 (PHQ-9), have proven to be effective, valid, and low-cost tools for identifying depressive symptoms in accordance with DSM-5. criterio (15, 16). This questionnaire has become one of the most widely used tools globally due to its brevity, ease of administration, and usefulness in resource-limited settings (17, 18).

In Latin America, various studies have supported the validity and reliability of the PHQ-9. In Colombia, among a university population, adequate internal consistencies were reported (α = 0.80; ω = 0.81) and concurrent validity with the HADS-D (ρ = 0.64) and PHQ-2 (ρ = 0.70) (9). In Peru, a large-scale population study confirmed a unidimensional structure with good fit (CFI = 0.936; RMSEA = 0.089; SRMR = 0.039), high reliability (α = ω = 0.87), and invariance by sex and age groups (19). In Argentina, high internal consistencies were observed (α = 0.87) and adequate diagnostic accuracy (AUC = 0.87) in primary care (20, 21). In Mexico and Chile, stable performance of the PHQ-9 has been documented in the general population and clinical samples, with good internal consistency and validity in local contexts (6, 22). In Puerto Rico, although two-dimensional and bifactor models were explored, the authors concluded that the most parsimonious interpretation of the scores is unidimensional, also confirming its invariance by sex (23). Additionally, in Spain, high internal consistency has been reported (ω = 0.89), good sensitivity (88%) and specificity (80%), as well as factorial stability in primary care; although both unifactorial and two-dimensional models showed acceptable fit (CFI = 0.95; RMSEA = 0.08), the high correlation between factors (r = 0.86) supported the parsimony of the unidimensional model, which also demonstrated invariance by sex, age, marital status, educational level, employment status, and over time (24).

Although some studies have explored alternative models to the unifactorial structure, including two-factor structures (25), bifactor models in Spanish-speaking university populations (21), and even four-factor solutions in community samples (26), the accumulated evidence indicates that such configurations tend to show very high correlations between factors and low reliability in the subfactors. The most recent meta-analysis (10) integrated the available evidence and confirmed that, although two-factor or bifactor models may show acceptable fit, the high correlation between factors and low subfactor reliability limit their usefulness; in contrast, the unidimensional model proves to be more parsimonious and stable, consolidating itself as the most robust option for clinical practice and cross-cultural research.

In this regard, the present study is based on the hypothesis that the PHQ-9 assesses a predominantly unidimensional construct, allowing the total score to be interpreted as a global measure of depressive symptoms and enabling the evaluation of its metric equivalence across countries and sexes. This is particularly relevant in Latin America and the Caribbean, where studies on the factorial invariance of the PHQ-9 remain scarce (16, 23). Therefore, the aim of this study was to assess the structural consistency and comparability of the PHQ-9 in a multinational sample of adults from the region, in order to provide evidence supporting its clinical and epidemiological use in diverse contexts.

## 2 Materials and method

### 2.1 Participants

The present study is part of a regional digital mental health monitoring strategy (27), coordinated across multiple countries in Latin America and the Caribbean. A non-probabilistic snowball sampling method was used, with a dissemination process structured in several stages. First, meetings were held with representatives of professional associations, educational, health, and community institutions, who were invited to participate and to disseminate the initiative within their networks. Subsequently, official invitations were sent through the human resources offices of universities, hospitals, clinics, and professional associations, accompanied by mass institutional emails. Finally, dissemination was carried out through institutional messaging groups (WhatsApp), managed by universities, hospitals, and professional associations, asking their members to share the invitation with their peers and local communities; this helped expand the coverage to new participants. This strategy combined formal and informal channels, reducing the risk of sample homogeneity and ensuring the inclusion of diverse profiles.

A self-administered online form was designed, available in linguistically and culturally adapted versions. The instrument included an introductory section with informed consent and guidelines to facilitate item comprehension. Dissemination was reinforced through local campaigns coordinated by technical teams previously trained in each participating country. Data collection was carried out between May 12, 2022, and November 27, 2023.

Initially, 14,842 participants were assessed; however, 2,718 cases were excluded for not completing the questionnaire, not accepting the informed consent, or selecting countries with low response volumes. The final sample consisted of 12,124 valid forms (54.32% women and 45.68% men), originating from Argentina (7.3%), Bolivia (6.7%), Colombia (10.3%), Chile (6.9%), Costa Rica (4.9%), El Salvador (5.7%), Ecuador (7.2%), Guatemala (4.7%), Panama (5.1%), Paraguay (5.7%), Peru (8.6%), Puerto Rico (5.8%), the Dominican Republic (6.6%), Uruguay (6.3%), and Venezuela (8.2%). The mean age was 31.14 years (SD = 18.78). The sample included professionals in health (12.5%), engineering and exact sciences (9.3%), social sciences (8.2%), legal, accounting, and administrative sciences (10.3%), education (11.4%), university students (14.2%), and individuals from the general community (34.1%). (see **Supplementary Participant Data**). In all included countries, the Spanish version of the PHQ-9 was administered. In the case of Puerto Rico, although it is legally a territory of the United States, Spanish is the predominant language spoken by the population, which justifies its use in this sample.

**Table 1** presents the sociodemographic distribution of the sample (N = 12,124) by country, mean age, age range, and sex. Overall, the sample reflected early and middle adulthood, with a slight female majority (54.3%).

**Table 1:** Descriptive statistics for the PHQ-9 items.

ítem	*M*	*SD*	Skew	Kurt.
1. Little interest or pleasure in doing things.	1.04	0.81	0.58	-0.01
2. Feeling down, depressed, or hopeless.	0.99	0.87	0.69	-0.09
3. Trouble falling or staying asleep or sleeping too much.	1.32	0.99	0.34	-0.90
4. Feeling tired or having little energy.	1.40	0.91	0.40	-0.66
5. Poor appetite or overeating.	1.30	1.00	0.32	-0.94
6. Feeling bad about yourself – or that you are a failure or have let yourself or your family down.	0.80	0.93	0.97	-0.02
7. Trouble concentrating on things, such as reading the newspaper or watching television.	0.96	0.88	0.71	-0.16
8. Moving or speaking so slowly that other people could have noticed. Or the opposite – being so fidgety or restless that you have been moving around a lot more than usual.	0.68	0.82	1.12	0.63
9. Thoughts that you would be better off dead or of hurting yourself in some way	0.28	0.63	2.51	6.20

### 2.2 Instrument

The Patient Health Questionnaire-9 (PHQ-9) was used, a self-report test designed to assess the presence and severity of depressive symptoms over the past two weeks. This instrument was developed by Spitzer et al. (28), as part of the Primary Care Evaluation of Mental Disorders (PRIME-MD). It consists of 9 items based on the DSM-5 diagnostic criteria for major depressive disorder, scored on a Likert scale from 0 (“not at all”) to 3 (“nearly every day”), with a total score ranging from 0 to 27. A cutoff score of ≥10 has demonstrated sensitivity and specificity greater than 80% in various studies. The PHQ-9 is a brief, sensitive, and easy-to-administer tool, with strong international empirical support confirming its unidimensionality, reliability, and cross-cultural equivalence across multiple countries and languages (29, 30). In this study, the Spanish version adapted for the general Spanish-speaking population was used (19, 23).

Additionally, for the analysis of concurrent validity, the Generalized Anxiety Disorder-7 (GAD-7) was used; it is a 7-item test that assesses generalized anxiety symptoms over the past two weeks. Each item is rated on a scale from 0 (“not at all”) to 3 (“nearly every day”), with a total score ranging from 0 to 21. A score of ≥10 is considered indicative of moderate to severe anxiety. The version used was validated in the general Spanish-speaking population by our group (López, N, et al., 2025; López, N, et al., 2025) (31) showing adequate validity and usefulness for screening in community settings.

Finally, Mini-Z 2.0 was used, a 10-item scale designed to assess burnout symptoms and associated psychosocial factors, including job satisfaction, workload, perceived control, stress, and alignment with institutional values. Item 1 specifically measures emotional exhaustion and is considered the primary indicator of burnout, with scores ≥3 suggesting clinical risk. Items are answered using either Likert or dichotomous formats, depending on their content. The version used was recently adapted and validated in a Spanish-speaking population (33). This instrument was applied only to participants who reported being actively employed in the online form; this condition was later randomly verified through confirmation emails. The purpose of its inclusion in this study was to examine the concurrent validity of the PHQ-9 in relation to burnout, a related but distinct construct from depression; in order to verify that the expected associations were maintained without compromising the specificity of the screening.

### 2.3 Data analysis

Data were digitized through the use of forms in a Google Sheets spreadsheet. The database was downloaded as an.xlsx file and imported into R software version 4.0.2, within the RStudio programming environment version 1.3.595 (34). The openxlsx package (35) was used for data import, tidyverse (36) and psych (37) for data preparation and analysis; lavaan (38), semPlot (39), and sem Tools (40) for confirmatory factor analysis (CFA) and measurement invariance; MBESS (41) for the calculation of the Omega coefficient and its confidence intervals, and WRS2 (42) for the computation of the winsorized correlation coefficient (tr. = 0.10). For the CFA, the robust Weighted Least Squares Mean and Variance adjusted (WLSMV) method was used as the estimator, and unidimensional structures were evaluated for both instruments. The following criteria were considered for assessing model fit: values ≥.90 and ≥.95 for the CFI and TLI were interpreted as acceptable and good fit, respectively; values ≤.08 and ≤.05 for the RMSEA as acceptable and good fit, respectively; and for the SRMR, values ≤.08 and ≤.06 were considered good and ideal fit, respectively. For the evaluation of measurement invariance, the procedure developed by Wu & Estabrook (43), was applied. Invariance assessment considered a sample size >300 and potential non-invariance was established when ΔCFI <.010, ΔTLI <.010, ΔSRMR <.030 y ΔRMSEA <.015 (44–46).

### 2.4 Ethical considerations

The study was conducted in accordance with the ethical principles established by the relevant institutional and national committees, as well as the guidelines of the Declaration of Helsinki (1975), updated in 2008. All participants provided informed consent through an online form prior to the start of the study. Upon completion of their participation, each received an individual report with their results, accompanied by relevant psychological and clinical recommendations. The research protocol was duly approved by the Ethics Committee of the Universidad de La Costa, under file No. 173 dated May 27, 2024, and registered under code INV.140-03-001-18.

## 3 Results

**Table 1** shows the descriptive statistics of the PHQ-9 items. Item 9 has the lowest mean value, while item 4 has the highest. Regarding skewness and kurtosis values, the vast majority are close to 0, with the exception of item 9, which presents the highest skewness and kurtosis values. Considering the content of this item, which reflects thoughts of self-harm, the high positive skewness and leptokurtic kurtosis indicate that the vast majority of respondents fall into the “not at all” category.

Confirmatory factor analysis was then conducted to examine the internal structure of the test. The models analyzed and their fit indices are presented in **Table 2**. For the PHQ-9, the unidimensional structure without correlated errors (Model 0) showed adequate fit indices according to the CFI, TLI, and SRMR; although the RMSEA presented a value close to the threshold of poor fit. Given the content overlap among somatic items (sleep–energy–appetite) and the modification indices, a minimal set of residual covariances was allowed (3, 4, 5). This decision is methodologically acceptable when local dependence is documented and there is theoretical justification (47, 48). Comparability by sex and country was assessed through multigroup CFA.

**Table 2:** Analyzed models and goodness-of-fit índices.

Modelos	χ²	gl	CFI	TLI	RMSEA	SRMR
Modelo 0	42,822.39	36	.978	.971	.098	.047
Model with covariance of error items: 4 and 5	42,350.15	35	.982	.976	.090	.043
Model with covariance of error items: 4 and 5, 3 and 4	41,975.62	34	.986	.980	.082	.039
Model with covariance of error items: 4 and 5, 3 and 4, 3 and 5	41,650.48	33	.989	.983	.075	.035

CFI, Comparative Fit Index; TLI, Tucker-Lewis Index; RMSEA, Root Mean Square Error of Approximation; SRMR, Standardized Root Mean Square Residual.

**Figure 1:**
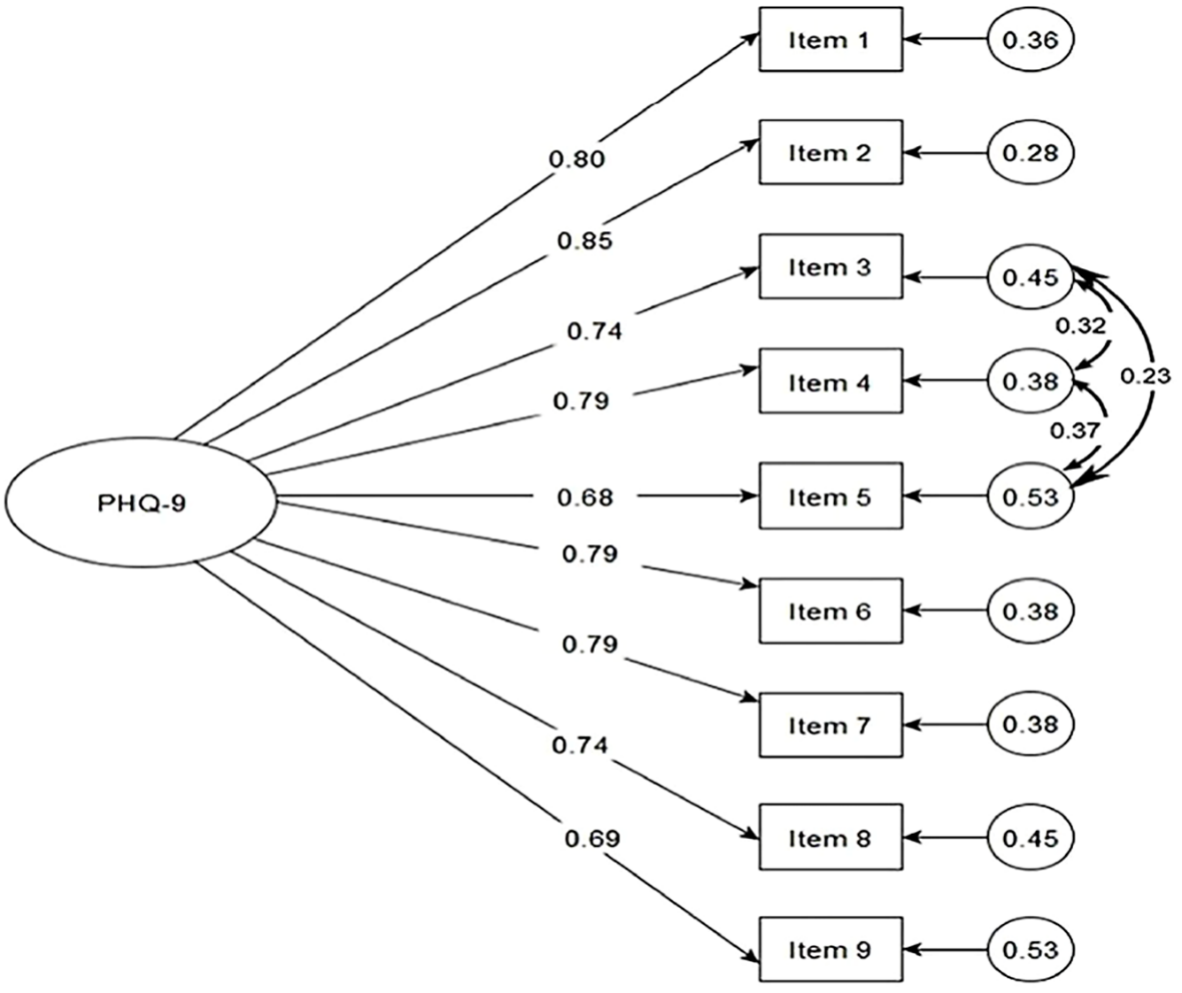
Factor loadings and correlated errors of the PHQ-9.

**Figure 1** presents the factor loadings of the model that showed the best fit for the PHQ-9. The factor loadings were greater than.68, and the values of the correlated errors ranged from.23 to.37.

In addition, the equivalence of the PHQ-9 factor structure was evaluated by sex and country. The results are presented in **Table 3**. In the measurement invariance analyses of the PHQ-9 factor structure, the fit indices ranged from good to adequate across all levels examined (configural, threshold, metric, scalar, and strict). Furthermore, the differences between the fit indices were below the threshold established in the data analysis section, indicating that the factor structure of the instrument demonstrates measurement invariance and is equivalent for both groups (men and women). The same applies when using country as the comparison variable. The structure (configural), thresholds, factor loadings (metric), intercepts (scalar), and residuals (strict) are equivalent across groups.

**Table 3:** Measurement invariance by sex and country.

	Model	X^2^	Gl	CFI	TLI	RMSEA	SRMR	ΔCFI	ΔTLI	ΔRMSEA	ΔSRMR
PHQ-9	Sex										
	Configural	538.029	48	.989	.983	.076	.037	–	–	–	–
	Threshold	586.372	57	.988	.985	.072	.037	-.001	.002	-.003	.000
	Metric	559.509	65	.989	.987	.065	.037	.001	.003	-.007	.000
	Scalar	540.200	73	.989	.989	.060	.037	.001	.002	-.005	.000
	Strict	561.721	82	.989	.990	.057	.039	.000	.001	-.003	.002
	Country										
	Configural	647.417	144	.989	.983	.077	.041	–	–	–	–
	Threshold	722.529	189	.988	.986	.069	.041	-.001	.003	-.008	.000
	Metric	685.194	229	.990	.990	.058	.041	.002	.004	-.011	.001
	Scalar	719.451	269	.990	.992	.053	.042	.000	.002	-.005	.001
	Strict	803.723	314	.989	.992	.051	.048	-.001	.001	.002	.006

Concurrent validity analysis was conducted by correlating the PHQ-9 with two measures related to depressive symptomatology: occupational burnout, assessed through the Mini-Z, and generalized anxiety symptoms, measured with the GAD-7. To interpret the correlations, the following thresholds were considered: r = .20 as weak, r = .50 as moderate, and.50–.80 as high (49). The results show a high positive correlation between the PHQ-9 and the GAD-7 (r = 0.792, p < 0.01), which supports that both instruments measure related but distinguishable constructs, as anxiety and depression often present comorbidly. Likewise, a high correlation was observed between the PHQ-9 and the Mini-Z (r = 0.635, p < 0.01), suggesting that high levels of occupational burnout are related to higher levels of depressive symptoms. (see **Supplementary Data 3**: Correlation Matrix).

Finally, McDonald’s Omega coefficient was used to assess the internal consistency of the instrument, yielding a value of 0.89, which indicates an adequate level of reliability.

## 4 Discussion

The findings of this study strengthen the psychometric evidence supporting the PHQ-9 as a clinically valid, robust, and culturally adaptable tool for assessing depressive symptoms across a broad sample of countries in Latin America and the Caribbean. Consistent with previous global research (50–52), our results confirm a unidimensional structure of the instrument, aligned with DSM-5 criteria. This configuration enables a concise and effective evaluation of depressive symptomatology without compromising diagnostic sensitivity.

The factorial stability of the unidimensional PHQ-9 model across the 15 included countries is particularly noteworthy. Despite cultural, linguistic, and socioeconomic differences among these contexts, the instrument demonstrated excellent fit, supporting its structural robustness and comparative utility across countries. This finding is consistent with multicultural studies that have consistently reported a predominant unidimensional structure (6, 21). However, some research has proposed alternative models with bifactorial or bidimensional configurations (23, 53). Although these approaches aim to capture structural variations in specific contexts, their results present relevant limitations.

In the study by Costantini et al. (53), the factor loadings associated with the specific factors were weak and inconsistent, which reduced the interpretability of the bifactor model. Similarly, Rosario-Hernández et al. (23) identified an acceptable fit for the two-dimensional model in Puerto Rican workers; however, the low loadings on the subfactors and the risk of overfitting limited its applicability, partly due to the reduced sociodemographic diversity of the sample. Other studies have also explored alternative models with heterogeneous results. Yu et al. (54) reported marginal fit for the unidimensional model (RMSEA = 0.122), which partially improved when allowing for error covariances; whereas Doi et al. (55), found that the bifactor model achieved superior fit indices (CFI = 0.980; RMSEA = 0.083) compared to the unidimensional model; although the subfactors showed weak loadings and the sample was recruited online without clinical diagnostic verification.

In contrast, our results consistently supported the unidimensional structure, reinforcing its practical utility for screening, without ruling out that alternative models may provide complementary information in specific contexts. Unlike the aforementioned studies, our work was based on a large and heterogeneous sample from Latin America and the Caribbean, which allowed us to confirm a stable unidimensional structure, with excellent fit and adequate cross-cultural validity. These findings are consistent with the most recent meta-analysis (10), which integrated evidence from 40 countries and demonstrated that, although bifactor or two-dimensional models may show statistically acceptable fit, the high correlations between factors and the low reliability of subfactors limit their practical usefulness.

Moreover, previous evidence indicates that the instrument with a unidimensional structure has demonstrated invariance across sociodemographic variables such as age, sex, educational level, and socioeconomic status (19). Therefore, the accumulated evidence supports the parsimony, stability, and invariance of the unidimensional model, confirming that the PHQ-9 should be interpreted primarily as a global measure of depressive symptoms. Thus, the alignment between our findings and the strongest international evidence helps close the conceptual debate, consolidating the unifactorial model as the most robust solution for research and screening in Latin America and the Caribbean.

It is worth noting that, like other studies using non-probabilistic sampling, our work is subject to the limitation of selection bias. However, unlike those investigations, the factorial structure and multigroup invariance were empirically evaluated using the dataset itself, which provides additional evidence of structural stability and comparability across countries and sexes. This approach partially mitigates concerns about the influence of such bias on the main results.

On the other hand, the high reliability coefficients observed both at the global level and within country and sex subgroups support the internal consistency of the PHQ-9 in LAC. These values exceed international standards established for brief screening scales (25, 56) and are consistent with studies conducted in various countries, including those in Asia, Europe, and the Americas (10, 14, 57, 58), The consistency of these results supports the psychometric stability of the instrument across diverse sociocultural contexts.

On the other hand, the multigroup invariance analysis represents a significant contribution. The results show that the PHQ-9 meets the criteria for configural, metric, and scalar invariance by both gender and country. These levels of invariance, which are not always achieved in comparative studies (59), allow for valid comparisons between groups and support its use in multicenter research. The ability to assess depressive symptoms equivalently across diverse populations is essential for advancing global mental health research and for the development of evidence-based public policies.

Compared to these findings, international evidence on the cultural invariance of the PHQ-9 remains limited in many regions. In Africa, for example (60),, found configural and metric invariance across samples from South Africa, Ghana, and Kenya, supporting its cross-cultural equivalence. Similarly, in rural areas of Asia (61),, reported a stable factor structure and good internal consistency in impoverished populations in India. In Europe (24),, documented invariance by age, sex, and educational level in a Spanish population.

These findings reinforce the need for culturally validated instruments in regions such as Latin America and the Caribbean, where mental health inequities and limited resource availability hinder access to timely diagnostic evaluations (22, 62). In this context, the PHQ-9 stands out as a useful tool due to its brevity, reliability, and ease of use-even in high-demand services with limited technical capacity. Its implementation is feasible in primary care, community programs, schools, correctional facilities, and public health campaigns, and it is particularly relevant for vulnerable populations such as migrants, individuals with chronic illnesses, workers exposed to stress, and rural communities, where early diagnosis and preventive interventions are a priority (63–65).

Beyond its clinical applicability, the confirmation of PHQ-9 invariance supports its usefulness for multicenter, longitudinal, and clinical studies in the region. This finding aligns with international evidence on its cross-cultural validity (51, 59) and is complemented by studies in Indigenous populations of Peru and Bolivia, where it has demonstrated strong properties in intercultural contexts (65, 66). The present work, along with previous research conducted by our team in clinical, general, and Indigenous samples (32, 67, 68), contributes to strengthening a regional diagnostic validation agenda aimed at mental health equity.

Finally, the results show that the PHQ-9 demonstrates concurrent validity with other scales measuring constructs related to depressive symptomatology, such as anxiety and occupational burnout. Its strong correlation with the GAD-7 replicates previous findings in multicultural contexts, which have documented consistent associations between depressive and anxious symptoms (69, 70). Complementarily, its moderate correlation with the Mini-Z supports its utility in identifying psychosocial distress in the workplace. In a study involving intensive care unit staff, Jackson et al. (71) reported that higher PHQ-9 scores were significantly associated with elevated levels of burnout (β = –0.32), confirming its sensitivity to occupational stress. Taken together, these findings reinforce the value of the PHQ-9 as a reliable tool for comprehensive mental health screening in clinical and workplace settings.

This study presents some limitations that should be considered when interpreting the findings. First, although participants from fifteen LAC countries were included, the sample is not representative of the entire region due to the non-probabilistic snowball sampling method. This limits the generalizability of the results to national or underrepresented rural populations. Nevertheless, the geographic breadth and sociodemographic heterogeneity of the sample provide a robust framework for regional interpretations. Likewise, the use of digital self-report sampling may have biased the sample toward individuals with greater technological access, higher educational levels, urban residence, and recruitment channels that may have varied between countries. This type of selection bias has also been noted in previous studies, reinforcing the need to interpret it as a shared limitation in the literature. While these characteristics limit population representativeness, the relative homogeneity of the sample, particularly regarding educational level and language proficiency, may have contributed to the internal consistency and invariance observed. Therefore, the diversity achieved and the robustness of the findings should be interpreted as evidence of internal validity within this sample, and their generalization to broader or less educated populations should be made with caution.

Second, the cross-sectional design prevents establishing causal relationships and assessing the temporal stability of the PHQ-9 or its sensitivity to change. These limitations could be addressed through longitudinal studies that examine its structural consistency and responsiveness to interventions. Additionally, the use of self-report measures may introduce biases, such as social desirability or comprehension difficulties, especially in contexts with low health literacy. Nevertheless, the brief format and accessible language of the PHQ-9 help mitigate these risks. Likewise, no formal diagnostic verification through clinical interviews was included, as the main objective was to assess the metric equivalence of the PHQ-9 across different countries and genders. The use of standardized self-reports such as the PHQ-9, which has been widely validated in population-based and cross-cultural studies, is an accepted strategy in this type of research. However, future studies should incorporate structured diagnostic interviews to complement self-reports and strengthen the clinical validity of the findings.

Third, although invariance by sex and country was confirmed, other dimensions of cultural diversity such as Indigenous populations, people of African descent, migrants, or individuals with low educational attainment were not explored. These groups require specific studies to assess the psychometric and cultural equivalence of the instrument.

Future research should incorporate qualitative or mixed-method approaches to explore the subjective meaning of the items, especially in contexts where somatic expressions of emotional distress are predominant. It is also essential to examine the instrument’s performance in various clinical settings, such as primary care, prisons, rural communities, and populations with chronic illnesses.

The present study provides robust evidence on the structural validity, internal consistency, concurrent validity, and factorial invariance of the PHQ-9 in Latin America and the Caribbean. The results confirm a unidimensional structure with excellent fit, high reliability, and equivalence by sex and country, supporting its use as a comparative instrument in multicenter and transnational contexts. These findings consolidate the PHQ-9 as a psychometrically sound and culturally adaptable tool for assessing depressive symptoms in diverse populations across the region.

## Data Availability

The raw data supporting the conclusions of this article will be made available by the authors, without undue reservation.

## Supplementary material

Supplementary Table 1 Demographic information of the participants.

Supplementary Table 2 Occupational distribution of participants by country.

Supplementary Table 3 Partial correlations of the PHQ-9 with Mini-Z by country.

## References

[1] Bada W Villacorta YR Novoa-Pallares O Torres A Gonzalez C Wisum U . Effectiveness of the PHQ-9 for detecting depressive symptoms in Peruvian Amazonian indigenous people. Gen Hosp Psychiatry. (2025) 97:1–2. doi: 10.1016/j.genhosppsych.2025.08.014, PMID: 40911944

[2] American Psychiatric Association . Diagnostic and Statistical Manual of Mental Disorders. American Psychiatric Association, Washington, DC, United States (2013).

[3] Organización Mundial De La Salud (OMS) . Depresión (2023). Available online at: https://www.who.int/es/news-room/fact-sheets/detail/depression (Accessed July 14, 2025).

[4] Salari N Hosseinian-Far A Jalali R Vaisi-Raygani A Rasoulpoor S Mohammadi M . Prevalence of stress, anxiety, depression among the general population during the COVID-19 pandemic: a systematic review and meta-analysis. Global Health. (2020) 16:57. doi: 10.1186/s12992-020-00589-w, PMID: 32631403 PMC7338126

[5] Mahmud S Mohsin M Dewan M Muyeed A . The global prevalence of depression, anxiety, stress, and insomnia among general population during COVID-19 pandemic: A systematic review and meta-analysis. Trends Psychol. (2022) 31:143–70. doi: 10.1007/s43076-021-00116-9, PMID: 40477944 PMC8726528

[6] Errazuriz A Beltrán R Torres R Passi-Solar A . The validity and reliability of the PHQ-9 and PHQ-2 on screening for major depression in spanish speaking immigrants in Chile: A cross-sectional study. Int J Environ Res Public Health. (2022) 19:13975. doi: 10.3390/ijerph192113975, PMID: 36360856 PMC9655214

[7] Chen XD Li F Zuo H Zhu F . Trends in prevalent cases and disability-adjusted life-years of depressive disorders worldwide: findings from the global burden of disease study from 1990 to 2021. Depress Anxiety. (2025) 2025. doi: 10.1155/da/5553491, PMID: 40313474 PMC12045679

[8] Han SS Zhang YS Zhu W Ye YP Li YX Meng SQ . Status and epidemiological characteristics of depression and anxiety among Chinese university students in 2023. BMC Public Health. (2025) 25:1189. doi: 10.1186/s12889-025-22443-7, PMID: 40155930 PMC11954289

[9] Cassiani-Miranda CA Cuadros-Cruz AK Torres-Pinzón H Scoppetta O Pinzón-Tarrazona JH López-Fuentes WY . Validez del Cuestionario de salud del paciente-9 (PHQ-9) para cribado de depresión en adultos usuarios de Atención Primaria en Bucaramanga, Colombia. Rev Colomb Psiquiatr. (2021) 50:11–21. doi: 10.1016/j.rcp.2019.09.001, PMID: 33648690

[10] Chae D Lee J Lee EH . Internal structure of the patient health questionnaire-9: A systematic review and meta-analysis. Asian Nurs Res (Korean Soc Nurs Sci). (2025) 19:1–12. doi: 10.1016/j.anr.2024.12.005, PMID: 39725053

[11] Chen C Meier ST . Burnout and depression in nurses: A systematic review and meta-analysis. Int J Nurs Stud. (2021) 124:104099. doi: 10.1016/j.ijnurstu.2021.104099, PMID: 34715576

[12] Caldichoury-Obando N Ripoll-Córdoba D Morales-Asencio B Ibañez-Reyes S Flórez Y Reyes-Cervantes C . Burnout en profesionales sanitarios de América Latina durante la pandemia de COVID-19. Rev Colomb Psiquiatr. (2024). doi: 10.1016/j.rcp.2024.04.008

[13] Schleef J Castellanos-Alvarenga LM Olivera MP Ortiz MS . Disentangling between-person and within-person associations of physical symptoms of depression with self-perceived health and life satisfaction: A longitudinal study in Chilean adults. J Health Psychol. (2024) 29:1377–89. doi: 10.1177/13591053241229533, PMID: 38433616

[14] Moreno-Agostino D Chua KC Peters TJ Scazufca M Araya R . Psychometric properties of the PHQ-9 measure of depression among Brazilian older adults. Aging Ment Health. (2022) 26:2285–90. doi: 10.1080/13607863.2021.1963951, PMID: 34409909

[15] Kroenke K Spitzer RL Williams JBW . The PHQ-9: validity of a brief depression severity measure. J Gen Intern Med. (2001) 16:606–13. doi: 10.1046/j.1525-1497.2001.016009606.x, PMID: 11556941 PMC1495268

[16] Rufino JV Rodrigues R Birolim MM Girotto E Mesas AE Martínez-Vizcaíno V . Analysis of the dimensional structure of the Patient Health Questionnaire-9 (PHQ-9) in undergraduate students at a public university in Brazil. J Affect Disord. (2024) 349:158–64. doi: 10.1016/j.jad.2024.01.051, PMID: 38199387

[17] Aslan J Cova F Saldivia S Bustos C Inostroza C Rincón P . Psychometric properties of the patient health questionnaire-9 in elderly Chilean primary care users. Front Psychiatry. (2020) 11. doi: 10.3389/fpsyt.2020.555011, PMID: 33312135 PMC7704434

[18] Cumbe VFJ Muanido A Manaca MN Fumo H Chiruca P Hicks L . Validity and item response theory properties of the Patient Health Questionnaire-9 for primary care depression screening in Mozambique (PHQ-9-MZ). BMC Psychiatry. (2020) 20:382. doi: 10.1186/s12888-020-02772-0, PMID: 32698788 PMC7374823

[19] Villarreal-Zegarra D Copez-Lonzoy A Bernabé-Ortiz A Melendez-Torres GJ Bazo-Alvarez JC . Valid group comparisons can be made with the Patient Health Questionnaire (PHQ-9): A measurement invariance study across groups by demographic characteristics. PloS One. (2019) 14:e0221717. doi: 10.1371/journal.pone.0221717, PMID: 31498796 PMC6733536

[20] Urtasun M Daray FM Teti GL Coppolillo F Herlax G Saba G . Validation and calibration of the patient health questionnaire (PHQ-9) in Argentina. BMC Psychiatry. (2019) 19:291. doi: 10.1186/s12888-019-2262-9, PMID: 31533674 PMC6751851

[21] López-Guerra VM López-Núñez C Vaca-Gallegos SL Torres-Carrión PV . Psychometric properties and factor structure of the patient health questionnaire-9 as a screening tool for depression among Ecuadorian college students. Front Psychol. (2022) 13. doi: 10.3389/fpsyg.2022.813894, PMID: 35572338 PMC9105228

[22] Arrieta J Aguerrebere M Raviola G Flores H Elliott P Espinosa A . Validity and utility of the patient health questionnaire (PHQ)-2 and PHQ-9 for screening and diagnosis of depression in rural chiapas, Mexico: A cross-sectional study. J Clin Psychol. (2017) 73:1076–90. doi: 10.1002/jclp.22390, PMID: 28195649 PMC5573982

[23] Rosario-Hernández E Rovira-Millán LV Merino-Soto C Angulo-Ramos M . Review of the psychometric properties of the Patient Health Questionnaire-9 (PHQ-9) Spanish version in a sample of Puerto Rican workers. Front Psychiatry. (2023) 14. doi: 10.3389/fpsyt.2023.1024676, PMID: 36865076 PMC9971011

[24] González-Blanch C Medrano LA Muñoz-Navarro R Ruíz-Rodríguez P Moriana JA Limonero JT . Factor structure and measurement invariance across various demographic groups and over time for the PHQ-9 in primary care patients in Spain. PloS One. (2018) 13:e0193356. doi: 10.1371/journal.pone.0193356, PMID: 29474410 PMC5825085

[25] Lamela D Soreira C Matos P Morais A . Systematic review of the factor structure and measurement invariance of the patient health questionnaire-9 (PHQ-9) and validation of the Portuguese version in community settings. J Affect Disord. (2020) 276:220–33. doi: 10.1016/j.jad.2020.06.066, PMID: 32697702

[26] Tseng VWS Tharp JA Reiter JE Ferrer W Hong DS Doraiswamy PM . Identifying a stable and generalizable factor structure of major depressive disorder across three large longitudinal cohorts. Psychiatry Res. (2024) 333:115702. doi: 10.1016/j.psychres.2023.115702, PMID: 38219346 PMC12165323

[27] Hinz A Klein AM Brähler E Glaesmer H Luck T Riedel-Heller SG . Psychometric evaluation of the Generalized Anxiety Disorder Screener GAD-7, based on a large German general population sample. J Affect Disord. (2017) 210:338–44. doi: 10.1016/j.jad.2016.12.012, PMID: 28088111

[28] Spitzer RL . Validation and utility of a self-report version of PRIME-MD;The PHQ primary care study. JAMA. (1999) 282:1737. doi: 10.1001/jama.282.18.1737, PMID: 10568646

[29] Bianchi R Verkuilen J Toker S Schonfeld IS Gerber M Brähler E . Is the PHQ-9 a unidimensional measure of depression? A 58,272-participant study. Psychol Assess. (2022) 34:595–603. doi: 10.1037/pas0001124, PMID: 35357877

[30] Zhou Y Xu J Rief W . Are comparisons of mental disorders between Chinese and German students possible? An examination of measurement invariance for the PHQ-15, PHQ-9 and GAD-7. BMC Psychiatry. (2020) 20:480. doi: 10.1186/s12888-020-02859-8, PMID: 33004042 PMC7531122

[31] López N Coronado JC Ripoll-Córdoba D Caldichoury N Quispe-Ayala C Morales-Asencio B . Factorial invariance of the generalized anxiety disorder scale (GAD-7) in Latin America and the Caribbean. Front Psychiatry. (2025) 16. doi: 10.3389/fpsyt.2025.1529424, PMID: 39950169 PMC11821638

[32] López N Morales-Asencio B Ripoll-Córdoba D Coronado-López J Caldichoury N Quispe-Ayala C . Internal validity and reliability of the GAD-7 test in latin america. Chronic Stress (Thousand Oaks). (2025) 9:24705470251315260. doi: 10.1177/24705470251315260, PMID: 40079046 PMC11898021

[33] Rivera-Fernández C Soto-Añari M Camargo L Caldichoury N Ramos L Porto MF . Validación del Mini-Z para la detección de burnout en personal sanitario Colombiano. Rev Colomb Psiquiatr. (2023) 52:S132–3. doi: 10.1016/j.rcp.2021.04.002, PMID: 34049683 PMC8149175

[34] RStudio Team . RStudio: Integrated Development Environment for R (2020). Boston, MA: RStudio, PBC. Available online at: http://www.rstudio.com (Accessed July 15, 2025).

[35] Schauberger P Walker A . openxlsx: Read, Write and Edit xlsx Files (2014). CRAN: Contributed Packages. Available online at: https://CRAN.R-project.org/package=openxlsx accessed on July 15, 2025.

[36] Wickham H Averick M Bryan J Chang W McGowan L François R . Welcome to the Tidyverse. J Open Source Softw. (2019) 4:1686. doi: 10.21105/joss.01686

[37] Revelle W . psych: Procedures for personality and psychological research. R package version 2.0. 2020.

[38] Rosseel Y . lavaan: an *R* package for structural equation modeling. J Stat Softw. (2012) 48. doi: 10.18637/jss.v048.i02

[39] Epskamp S Stuber S Nak J Veenman M Lemmens M . semPlot: Path diagrams and visual analysis of various SEM packages’ output. (2019). R package version 1.1.

[40] Jorgensen TD Pornprasertmanit S Schoemann AM Rosseel Y . semTools: Useful tools for structural equation modeling. (2020). R package version 0.5-3.

[41] Kelley K . MBESS: Methods for the Behavioral, Educational, and Social Sciences (2020). Available online at: https://rdrr.io/cran/MBESS/ (Accessed July 15, 2025). R package version 4.9.3.

[42] Mair P Wilcox R . Robust statistical methods in R using the WRS2 package. Behav Res Methods. (2020) 52:464–88. doi: 10.3758/s13428-019-01246-w, PMID: 31152384

[43] Wu H Estabrook R . Identification of confirmatory factor analysis models of different levels of invariance for ordered categorical outcomes. Psychometrika. (2016) 81:1014–45. doi: 10.1007/s11336-016-9506-0, PMID: 27402166 PMC5458787

[44] Chen FF . Sensitivity of goodness of fit indexes to lack of measurement invariance. Struct Equ Modeling. (2007) 14:464–504. doi: 10.1080/10705510701301834

[45] Sánchez-Álvarez N Extremera-Pacheco N Rey L Chang EC Chang OD . Frequency of suicidal ideation inventory: psychometric properties of the spanish version. Psicothema. (2020) 32:253–60. doi: 10.7334/psicothema2019.344, PMID: 32249752

[46] Svetina D Rutkowski L Rutkowski D . Multiple-Group Invariance with Categorical Outcomes Using Updated Guidelines: An Illustration Using M *plus* and the lavaan/semTools Packages. Struct Equ Modeling. (2020) 27:111–30. doi: 10.1080/10705511.2019.1602776

[47] Hermida R . The problem of allowing correlated errors in structural equation modeling: Concerns and considerations. Comput Methods Soc Sci. (2015) 3:5–17.

[48] Brown TA . Confirmatory factor analysis for applied research. New York: Guilford Publications (2015).

[49] Cohen J . Statistical power analysis for the behavioral sciences. Routledge, New York, United States (2013).

[50] Sun Y Kong Z Song Y Liu J Wang X . The validity and reliability of the PHQ-9 on screening of depression in neurology: a cross sectional study. BMC Psychiatry. (2022) 22:98. doi: 10.1186/s12888-021-03661-w, PMID: 35139810 PMC8827244

[51] Smith ML Sanchez SE Rondon M Gradus JL Gelaye B . Validation of the patient health Questionnaire-9 (PHQ-9) for detecting depression among pregnant women in Lima, Peru. Curr Psychol. (2022) 41:3797–805. doi: 10.1007/s12144-020-00882-2, PMID: 35757832 PMC9216168

[52] Di Matteo R Bolgeo T Simonelli N Dal Molin A Bassola B Lusignani M . Psychometric properties and measurement invariance of the patient health questionnaire 9 in an italian coronary heart disease population. J Cardiovasc Nurs. (2025). doi: 10.1097/JCN.0000000000001178, PMID: 39932691

[53] Costantini L Pasquarella C Odone A Colucci ME Costanza A Serafini G . Screening for depression in primary care with Patient Health Questionnaire-9 (PHQ-9): A systematic review. J Affect Disord. (2021) 279:473–83. doi: 10.1016/j.jad.2020.09.131, PMID: 33126078

[54] Yu X Tam WWS Wong PTK Lam TH Stewart SM . The Patient Health Questionnaire-9 for measuring depressive symptoms among the general population in Hong Kong. Compr Psychiatry. (2012) 53:95–102. doi: 10.1016/j.comppsych.2010.11.002, PMID: 21193179

[55] Doi S Ito M Takebayashi Y Muramatsu K Horikoshi M . Factorial validity and invariance of the Patient Health Questionnaire (PHQ)-9 among clinical and non-clinical populations. PloS One. (2018) 13:e0199235. doi: 10.1371/journal.pone.0199235, PMID: 30024876 PMC6053131

[56] Wang Y Liang L Sun Z Liu R Wei Y Qi S . Factor structure of the patient health questionnaire-9 and measurement invariance across gender and age among Chinese university students. Medicine. (2023) 102:e32590. doi: 10.1097/MD.0000000000032590, PMID: 36607886 PMC9829284

[57] Borgogna NC Brenner RE McDermott RC . Sexuality and gender invariance of the PHQ-9 and GAD-7: Implications for 16 identity groups. J Affect Disord. (2021) 278:122–30. doi: 10.1016/j.jad.2020.09.069, PMID: 32956961

[58] Dajpratham P Pukrittayakamee P Atsariyasing W Wannarit K Boonhong J Pongpirul K . The validity and reliability of the PHQ-9 in screening for post-stroke depression. BMC Psychiatry. (2020) 20:291. doi: 10.1186/s12888-020-02699-6, PMID: 32517743 PMC7285729

[59] Harry ML Coley RY Waring SC Simon GE . Evaluating the cross-cultural measurement invariance of the PHQ-9 between American Indian/Alaska Native adults and diverse racial and ethnic groups. J Affect Disord Rep. (2021) 4:100121. doi: 10.1016/j.jadr.2021.100121, PMID: 34142103 PMC8208497

[60] Makhubela M Khumalo IP . Psychometric evaluation of the PHQ-9 in university students: Factorial validity and measurement equivalence across three African countries. Curr Psychol. (2023) 42:18061–9. doi: 10.1007/s12144-022-02997-0

[61] De Man J Absetz P Sathish T Desloge A Haregu T Oldenburg B . Are the PHQ-9 and GAD-7 suitable for use in India? A psychometric analysis. Front Psychol. (2021) 12. doi: 10.3389/fpsyg.2021.676398, PMID: 34054677 PMC8155718

[62] Shaff J Kahn G Wilcox HC . An examination of the psychometric properties of the Patient Health Questionnaire-9 (PHQ-9) in a Multiracial/ethnic population in the United States. Front Psychiatry. (2024) 14. doi: 10.3389/fpsyt.2023.1290736, PMID: 38293592 PMC10824969

[63] Na PJ Yaramala SR Kim JA Kim H Goes FS Zandi PP . The PHQ-9 Item 9 based screening for suicide risk: a validation study of the Patient Health Questionnaire (PHQ)–9 Item 9 with the Columbia Suicide Severity Rating Scale (C-SSRS). J Affect Disord. (2018) 232:34–40. doi: 10.1016/j.jad.2018.02.045, PMID: 29477096

[64] Wisting L Johnson SU Bulik CM Andreassen OA Rø Ø Bang L . Psychometric properties of the Norwegian version of the Patient Health Questionnaire-9 (PHQ-9) in a large female sample of adults with and without eating disorders. BMC Psychiatry. (2021) 21:6. doi: 10.1186/s12888-020-03013-0, PMID: 33402149 PMC7786911

[65] Cjuno J Julca-Guerrero F Oruro-Zuloaga Y Cruz-Mendoza F Auccatoma-Quispe A Gómez Hurtado H . Adaptación cultural al Quechua y análisis psicométrico del Patient Health Questionnaire PHQ-9 en población Peruana. Rev Peru Med Exp Salud Publ. (2023) 40:267–77. doi: 10.17843/rpmesp.2023.403.12571, PMID: 37991030 PMC10959517

[66] Bazo-Alvarez JC Aparicio ARO Robles-Mariños R Julca-Guerrero F Gómez H Bazo-Alvarez O . Cultural adaptation to Bolivian Quechua and psychometric analysis of the Patient Health Questionnaire PHQ-9. BMC Public Health. (2024) 24:129. doi: 10.1186/s12889-023-17566-8, PMID: 38195478 PMC10775527

[67] López N Coronado JC Quispe-Ayala C García-Roncallo P Cárdenas-Valverde J Flórez Y . Clinical validation of the GAD-7 for the Peruvian Quechua population. Gen Hosp Psychiatry. (2025) 92:119–20. doi: 10.1016/j.genhosppsych.2024.10.008, PMID: 39426925

[68] Caldichoury N Quispe-Ayala C Coronado JC Castellanos-Alvarenga LM Salazar D Morales-Asencio B . Clinical utility of the GAD-7 for detecting generalized anxiety in Quechua indigenous people. Front Psychiatry. (2025) 16. doi: 10.3389/fpsyt.2025.1565895, PMID: 40521593 PMC12163617

[69] Odero SA Mwangi P Odhiambo R Mumbua Nzioka B Shumba C Ndirangu-Mugo E . Psychometric evaluation of PHQ–9 and GAD–7 among community health volunteers and nurses/midwives in Kenya following a nation-wide telephonic survey. Front Psychiatry. (2023) 14. doi: 10.3389/fpsyt.2023.1123839, PMID: 37324823 PMC10264862

[70] Steinman L Phal O Srou R Ung K LoGerfo J Veith RC . Improving recognition of common mental health disorders in Cambodia: Validation of the PHQ-9 and GAD-7 and development of a brief mental health screener. PloS Ment Health. (2025) 2:e0000228. doi: 10.1371/journal.pmen.0000228 PMC12798203 41661885

[71] Jackson JL Kuriyama A Muramatsu K . A model of burnout among healthcare professionals. J Gen Intern Med. (2024) 39:373–6. doi: 10.1007/s11606-023-08514-8, PMID: 37946016 PMC10897092

