# Supplementary material for: Factorial invariance of the Spanish version of the PHQ-9 by gender and country in Latin America and the Caribbean
Source: Front Psychiatry. 2025 Nov 19;16:1667612. doi: 10.3389/fpsyt.2025.1667612 (PMC12673220; doi:10.3389/fpsyt.2025.1667612)
Supplement: Supplementary Table 1 — Demographic information of the participants. [file DataSheet1.docx]

**Complementary data table 1: Demographic information of the participants**

| **Countries** | **No. subjects** | **%** | **Age** | | **Age ranges** | | **% by gender** | |
| --- | --- | --- | --- | --- | --- | --- | --- | --- |
|  |  |  | **M** | **SD** | **Lower Limit** | **Upper Superior** | **Male** | **Female** |
| Argentina | 885 | 7,3 | 30,53 | 7,18 | 21,78 | 39,31 | 49,96 | 50,04 |
| Bolivia | 812 | 6,7 | 34,28 | 9,25 | 25,5 | 43,06 | 43,74 | 56,26 |
| Colombia | 1249 | 10,3 | 33,54 | 10,21 | 24,76 | 42,32 | 48,68 | 51,32 |
| Chile | 837 | 6,9 | 30,54 | 9,62 | 21,76 | 39,32 | 47,73 | 52,27 |
| Costa Rica | 594 | 4,9 | 29,36 | 7,34 | 20,58 | 38,14 | 43,7 | 56,3 |
| El Salvador | 691 | 5,7 | 39,03 | 7,31 | 30,25 | 47,81 | 44,68 | 55,32 |
| Ecuador | 873 | 7,2 | 32,68 | 10,37 | 23,9 | 41,46 | 43,75 | 56,25 |
| Guatemala | 570 | 4,7 | 34,3 | 6,26 | 25,52 | 43,08 | 53,16 | 53,16 |
| Panamá | 618 | 5,1 | 31,05 | 11,2 | 22,27 | 39,83 | 45,77 | 54,23 |
| Paraguay | 691 | 5,7 | 29,38 | 9,08 | 20,6 | 38,16 | 43,99 | 56,01 |
| Perú | 1042 | 8,6 | 27,62 | 9,45 | 18,84 | 36,4 | 43,67 | 55,33 |
| Puerto Rico | 703 | 5,8 | 26,5 | 6,05 | 17,72 | 35,28 | 47,34 | 52,66 |
| R. Dominicana | 800 | 6,6 | 28,27 | 9,04 | 19,49 | 37,05 | 44,69 | 55,31 |
| Uruguay | 764 | 6,3 | 32,44 | 10,19 | 23,66 | 41,22 | 46,92 | 53,08 |
| Venezuela | 995 | 8,2 | 27,61 | 9,16 | 18,83 | 36,39 | 42,74 | 57,26 |
| Total | 12124 | 100 | 31,14 | 8,781 |  |  | 46,03 | 54,32 |

**Supplementary Table 3: Partial correlations of the PHQ-9 with Mini-Z by country**

| **Countries** | **GAD-7 (r)** | **Mini-Z (r)** | **p-value** |
| --- | --- | --- | --- |
| Argentina | 0.802 | 0.649 | < 0.01 |
| Bolivia | 0.798 | 0.669 | < 0.01 |
| Colombia | 0.802 | 0.714 | < 0.01 |
| Chile | 0.822 | 0.705 | < 0.01 |
| Costa Rica | 0.781 | 0.623 | < 0.01 |
| El Salvador | 0.780 | 0.621 | < 0.01 |
| Ecuador | 0.824 | 0.689 | < 0.01 |
| Guatemala | 0.791 | 0.637 | < 0.01 |
| Panama | 0.783 | 0.719 | < 0.01 |
| Paraguay | 0.803 | 0.745 | < 0.01 |
| Peru | 0.795 | 0.699 | < 0.01 |
| Puerto Rico | 0.797 | 0.605 | < 0.01 |
| R. Dominicana | 0.755 | 0.621 | < 0.01 |
| Uruguay | 0.811 | 0.705 | < 0.01 |
| Venezuela | 0.805 | 0.617 | < 0.01 |
| Total | 0.792 | 0.635 | < 0.01 |

**Complementary data table 2: Occupational distribution of participants by country**

| **Pais** | **Health** | **Engineering and Exact Sciences** | **Social Sciences** | **Legal, Accounting and Administrative Sciences** | **Education** | **University Students** | **Community** | **Total** |
| --- | --- | --- | --- | --- | --- | --- | --- | --- |
| Argentina | 111 | 82 | 73 | 91 | 101 | 126 | 301 | 885 |
| Bolivia | 102 | 76 | 67 | 84 | 93 | 115 | 275 | 812 |
| Colombia | 156 | 116 | 102 | 129 | 142 | 177 | 427 | 1249 |
| Chile | 105 | 78 | 69 | 86 | 95 | 119 | 285 | 837 |
| Costa Rica | 74 | 55 | 49 | 61 | 68 | 84 | 203 | 594 |
| El Salvador | 86 | 64 | 57 | 71 | 79 | 98 | 236 | 691 |
| Ecuador | 109 | 81 | 72 | 90 | 100 | 124 | 297 | 873 |
| Guatemala | 71 | 53 | 47 | 59 | 65 | 81 | 194 | 570 |
| Panama | 77 | 57 | 51 | 64 | 70 | 88 | 211 | 618 |
| Paraguay | 86 | 64 | 57 | 71 | 79 | 98 | 236 | 691 |
| Peru | 130 | 97 | 85 | 107 | 119 | 148 | 356 | 1042 |
| Puerto Rico | 88 | 65 | 58 | 72 | 80 | 100 | 240 | 703 |
| R. Dominicana | 100 | 74 | 66 | 82 | 91 | 114 | 273 | 800 |
| Uruguay | 96 | 71 | 63 | 79 | 87 | 108 | 260 | 764 |
| Venezuela | 124 | 93 | 82 | 102 | 113 | 141 | 340 | 995 |
| Total: | 1515 | 1126 | 998 | 1248 | 1382 | 1721 | 4134 | 12124 |
| Total % | 12,5% | 9,3% | 8,2% | 10,3% | 11,4% | 14,2% | 34,1% | 100% |
